# An evidence-based approach to understanding the competency development needs of the health service management workforce in Australia

**DOI:** 10.1186/s12913-018-3760-z

**Published:** 2018-12-18

**Authors:** Zhanming Liang, Felicity C. Blackstock, Peter F Howard, David S Briggs, Sandra G Leggat, Dennis Wollersheim, David Edvardsson, Aziz Rahman

**Affiliations:** 1 School of Psychology and Public Health, La Trobe University, Melbourne, Australia; 2 School of Science and Health, Western Sydney University, Building 24, Campbelltown Campus, Sydney, Australia; 3 College of Science, Health and Engineering, La Trobe University, Melbourne, Australia; 4 The Society of Health Administration Programs in Education, Armidale, Australia; 5 School of Nursing and Midwifery, La Trobe University, Melbourne, Australia

**Keywords:** Management competency, Competency development, Health service management workforce, Workforce strategies

## Abstract

**Background:**

Competent managers are essential to the productivity of organisations and the sustainability of health systems. Effective workforce development strategies sensitive to the current competency development needs of health service managers (HSMs) are required.

**Purpose:**

To conduct a 360° assessment of the competence of Australian HSMs to identify managerial competence levels, and training and development needs.

**Methods:**

Assessment of 93 middle-level HSMs from two public hospitals (*n* = 25) and five community health services (CHS) (*n* = 68), using the Managerial Competency Assessment Partnership (MCAP) framework and tool, conducted between 2012 and 2014 in Victoria, Australia.

**Results:**

Mean competency scores from both self- and combined colleagues’ assessments indicated competence (scores greater than five but less than six) without guidance, but many HSMs have not had extensive experience. Around 12% of HSMs were unable to demonstrate the competency of ‘*evidence-informed decision-making*’ and 4% of HSMs were unable to demonstrate the competency of ‘*enabling and managing change*’.

**Conclusion:**

The assessments confirmed managerial competence for the majority of middle-level HSMs from hospitals and CHS in Victoria, but found competency gaps. In addition, the assessment confirmed managerial strengths and weaknesses varied across management groups from different organisations. These findings suggest that the development of strategies to strengthen the health service management workforce should be multifaceted.

**Practice implications:**

A focus on competency in performance evaluation and development using the MCAP framework and tool not only provides insights into performance of HSMs, but also has the potential to provide an organisation strategic advantage through succession planning and advancing managers’ competence via learning needs analysis and targeted professional development. Linking competencies of HSMs to organisational objectives and strategies provides optimal use of the human resource capacity, improving the organisation’s productivity and sustainability.

## Background

Empirical evidence has demonstrated a link between the development and competence of managers and positive productivity of organisations [1, 2]. In healthcare, leadership styles and health services managerial development has demonstrated an impact on healthcare service provision, workforce and work environments [3, 4]. However, the Australian healthcare system is considered to be at risk of inadequate or even dangerous management practice [5] and poor management is one of the contributing factors to high turnover amongst healthcare professional staff [6]. The potential inadequacy in leadership and management of the senior executive team has been outlined by a number of official inquiries into the quality and safety scandals in the Australian public hospitals [7, 8].

Health services managers (HSM) may be inadequately equipped for their management responsibilities as the role of health service management has not been well defined in Australia and HSM are often recruited based on a clinicians’ seniority and clinical leadership rather than management competency and capability [9]. Although HSMs tended to possess postgraduate qualifications, such qualifications were largely non-management related, usually in the field of clinical practice of the appointed HSM. A recent study conducted in Victoria determined that more than half of the middle-level HSM, and more than 70% of the senior level HSM had been awarded postgraduate qualifications [10, 11]. However, less than 10% of these qualifications were management specific [10, 11]. This suggests that formal managerial development for personnel in healthcare is currently not a requirement of appointment to middle and senior level HSM positions in Australia, and raises questions on the managerial competency of personnel recruited to HSM roles. In contrast, certification of healthcare managers by a professional body is usual practice in the USA [12].

The importance of the training and education of HSMs to confront the complex and dynamic health systems is well recognised [9, 13], and empirical evidence strongly suggests that training is effective for managerial development [14]. Managerial professional development can be achieved through a number of mechanisms such as providing formal education through universities in the field of management; in-service training; in-house use of mentors and study groups; offsite intensive training, and seminars and conferences [15]. To facilitate managerial development, efforts may be seen in the form of investment in professional development activities by healthcare organisations; training and development opportunities provided by professional institutions and various non-formal educational bodies; short training courses provided in partnership between universities and professional institutions, and formal higher degree university programs. However, to progress the design of initiatives to support HSM managerial development, an understanding of current competency levels and learning needs is first required. To date, competency assessment has not been widely embedded into the performance management process of healthcare organisations where HSMs’ managerial competence is assessed regularly to inform the requirements for training and education. Therefore, unlike the USA [12], the management competency requirements and learning needs of HSMs in Australia are unclear.

A competency-based education framework has been widely applied to health service management training in many higher education institutions in the US and the UK [16, 17] and has been recommended by the Australian Institute of Health and Welfare as detailed in the National Health and Hospital Reform Commission (2009) report [5]. However, the application of such an education framework in Australia has been limited, most likely due to the lack of understanding of competency requirements for HSMs in Australia [10]. Analysis of the postgraduate health service management degrees offered by Australian universities confirms a high level of variation in the core foundation knowledge for managing health services [18]. A more recent analysis of the 13 Australian masters’ level programs in health administrations also confirmed that no consistent approach in considering the actual competency requirements of health service management workforce has been adopted in designing the existing health administration programs [19]. Although some of the programs are mapped against the capability framework provided by the Australasian College of Health Service Managers as part of the accreditation requirements, a competency-based approach has not yet been fully adopted. Potentially more effective workforce training and development strategies that are sensitive to the current competency development needs of HSMs [13] and more effective investment in the development of the competence of HSMs [13, 20, 21] can be implemented.

It has been recommended that competency-based assessment, education and training for HSMs be established and adopted in Australia [22]. In 2011, a Management Competency Assessment Partnership (MCAP) project was established in Victoria, Australia. The project developed and validated a leadership and management competency framework and a management competency assessment tool. The MCAP Leadership and Management Competency Framework (MCAP LMCF) was developed through a rigorous and staged process, adapting the frameworks and assessment processes described by Catano & Campbell, C. (2007) [23] and Garman, Tyler & Darnall (2004) [24]. The MCAP LMCF is purpose built for the healthcare setting and HSM competency assessment, consisting of the following six core management competencies that are measured by 79 behavioural items [11, 12, 24]:

- C1 Evidence-informed decision-making **(Evidence)**
- C2 Operations, administration and resource management (**Resources**)
- C3 Demonstrated knowledge of healthcare environment and the organisation (**Knowledge**)
- C4 Interpersonal, communication qualities and relationship management (**Communication**)
- C5 Leading people and organisations (**Leadership**)
- C6 Enabling and managing change (**Change**)

(Appendix provides details of these behavioural items)

Based on the MCAP LMCF, an online evidence-based management competency assessment tool (the MCAP Tool), comprising a 360° assessment (involving self, supervisor, colleague, and supervisee) was developed and validated in Victoria between 2012 and 2014 [11, 12, 24, 25].

The purpose of this paper is to examine the competence of middle-level HSMs in Victoria, using the MCAP 360° assessment, and to identify competency based training and education directions for health service managers by determining:

1. The managerial competencies of health service managers at individual and group levels;
2. The managerial strengths and areas for improvement of health service managers; and
3. Any variation of the competence of health service managers between different healthcare organisations and settings.

## Methods

This cross sectional descriptive study examined the competency levels and developmental needs of HSMs working in public hospitals and community health centres (CHS) in Victoria, Australia.

Ethics approval was obtained from the Human Research Ethics Committee of La Trobe University (HREC No. S15/75) prior to conducting the project. Consent to participate was sought from all participants online before the start of the competency assessment.

### Participants

Health service managers from two public hospitals and five CHS in Victoria, Australia, were invited to participate in the assessments. Middle-level managers from these organisations were targeted for recruitment, with no specific exclusion criteria. For the purposes of this study, middle-level managers were considered those employed in roles classified as Level 4 (L4) managers in public hospitals and Level 3 (L3) managers in CHS. In Australia, management level is defined according to the supervision structure, where the Chief Executive Officer is the Level I manager of the healthcare organisation. The participants are referred to as the primary participating managers (PPM). Supervisors (more senior managers), peers (managers from the same management level within the same organisation) and reports (staff who report directly to the primary participating managers) of the PPMs were then invited to conduct assessments on the PPM. The organisations involved in the study were a convenience sample of partner organisations of the MCAP project. The organisations represented metropolitan and regional sites to improve generalisability of results across the state.

### Competency assessment

The MCAP 360° subjective assessment included a self-assessment (completed by the PPMs themselves), a supervisor assessment, a peer assessment and a report assessment. A peer was a colleague working in the same area as the PPM, but not directly supervising or reporting to the PPM. The development of the MCAP assessment and identification of core managerial competencies for the healthcare sector is described in Liang et al. (2013a, 2013b) [11, 25]. The MCAP is a reliable and valid measure of HSM managerial competency [26].

Assessments were completed online using a web-based platform developed specifically by the research team for hosting and analysing all assessments. The web-based platform contained the MCAP Assessment Tool, previously outlined in Liang et al. (2017) [25]. To minimise entry error, the assessment process limited the numerical responses to each question and unanswered questions were labelled as missing. Questions relating to participants’ age, sex, years of management experience, educational backgrounds and professional memberships were also included in the subjective self-assessment to collect demographic information on the PPMs. Table 1 summarises the descriptors for the 7-point Likert scale used on each of the 79 behavioural items (Appendix) for the six competency areas of the MCAP assessment. For the purpose of this study, a score of five (competent, no guidance is required) or more is defined as ‘competent’. Scores less than five are defined as less than fully competent.

**Table 1:** Behavioural scale / levels for self- and colleague’s assessment

1	Not competent	Do not understand the requirement and am not capable of applying it in my role
2	Basic or novice	May be capable of demonstrating minor aspects in my role
3	Advanced beginner	May be capable of demonstrating in my role, but not in all required aspects
4	Competent but needs guidance occasionally	Can generally demonstrate in my role, but guidance is needed occasionally
5	Competent, no guidance is required	Can generally demonstrate in my role independently, but have not had extensive experience
6	Proficient	Always apply appropriately in my role, have had extensive experience
7	Superior expertise / skill coach for others	Always apply appropriately in my role, have had extensive experience and can teach this competency to others

### Data management and analysis

Raw data from the server hosting the website were downloaded into MS Excel files for consistency checking. The mean scores for each competency were calculated from the scores of the behavioural items, as were the ‘combined competency’ scores. In addition to the results for the four subjective assessment types (self, supervisor, peer and report), a ‘combined colleagues’ variable was calculated from the mean scores of the supervisor, peer and supervisee assessments to protect the confidentiality of the colleagues who completed the assessments on the PPM. In addition, item and competency scores were grouped into five levels (Not yet fully competent: < 3.0 and 3.0- < 4.0; Competent (requiring vs not requiring guidance): 4.0- < 5.0 and 5.0- < 6.0; Proficient or higher: 6.0–7.0). Scores less than 5.0 were considered less than competent. The data were then imported into into SPSS© version 22 for statistical analysis. Univariate analyses for both individuals and groups (management and organisational levels), and all demographic variables were completed. The distribution of continuous variables was checked for normality. Appropriate bivariate analyses to investigate the effect of possible competency predictors included age, sex, management level, number of years as a manager, education, professional membership and sector. The selection of tests depended on the distribution of the dependent variable (competency scores) and the independent variable type (continuous or categorical). In addition, t-tests or Mann-Whitney U tests were used to compare mean competency scores between the self-assessments and separately for the supervisor, peer and report assessments. Following this, multivariate analyses were performed using univariate analysis of variance and general linear modelling. These analyses were used for assessing the effects of multiple predictors (see above) of the mean competency scores. *P* values less than 0.05 were considered statistically significant.

The results are presented as mean scores for each behavioural item (79), the six core competencies and the combined competencies. The differences between the means of the supervisor and the self-assessment scores were calculated and analysed. The percentage of participants whose supervisors scored the participant greater or less than two standard deviations from the mean difference was calculated. In addition, the percentage of participants whose supervisors scored the participant greater or less than one standard deviation from the mean difference was calculated. Further details on score calculation for the MCAP are provided in Howard et al. (2017) [27].

## Results

Ninety-three middle-level managers from two public hospitals (*n* = 25) and five CHS (*n* = 68) participated in the assessments (85% of the managers invited). Three-hundred and nineteen of their colleagues participated in the 360° subjective assessment. Colleagues included 90 supervisors, 95 peers and 134 reports. One hospital and two CHS sites decided to invite two peers and two reports to complete the assessments for each PPM, leading to the greater number of peer and report assessments. Participation was comprehensive by PPMs completing the 360° subjective assessments, with a near 100% completion rate for the 79 behavioural items provided to test the six core competencies. Of the 5250 total items assessed by the MCAP 360° of all PPMs, only 3% of item numbers had missing data points.

### Demographic and educational background: Public hospitals

Of the 25 public hospital PPMs, 76% were female, and the average age of the group was 47 years. The median period for the PPMs in a similar position was 6.0 years. Eighty percent (20/25) of PPMs held postgraduate qualifications, of which 50% (10/20) were in health or business management. Fourteen of the 25 hospital managers (56%) had current professional membership associated with their clinical discipline. Only four of the 25 (16%) managers had current membership of the Australasian College of Health Service Managers (ACHSM) – the professional organisation for Australian HSMs. (See Table 2.)

**Table 2:** Demographic details of participants by sector

Sector	Hospital	CHS
Sex (% female)	76	77
Mean Age (years)	47.0	44.0
Years in similar position (mean)	6.0	5.0
Postgraduate Qualification (%)	20/25 (80)	37/67 (55)
Qualification in management (%)	10/20 (50)	15/37 (41)
Professional membership (%)	14/25 (56)	26/66 (38)
ACHSM membership (%)	4/25 (16)	1/67 (2)

### Demographic and educational background: CHS

Of the 68 CHS PPMs, 77% were female with an average age of the group of 44 years. The median period for the managers in a similar position was 5.0 years. Fifty-five percent (37/68) of PPMs held postgraduate qualifications, of which 41% (15/37) were in health or business management. Twenty-six of the CHS PPMs (38%) had maintained professional membership associated with their clinical discipline, and only one PPM had maintained membership of ACHSM. Table 2 summarises these results.

### Overall competency scores for middle-level HSMs

Table 3 shows the mean ‘combined competency’ scores from the self-assessment (SA) and combined-colleague assessment (CCA) for PPMs, presented by sector and assessment types. Both the SA and CCA recorded mean scores were > 5 for both hospital and CHS managers. There were no statistically significant differences between the SA competency means and CCA competency means between sectors. However, the CCA scores were all significantly higher than the SA scores for both sectors (Mann-Whitney U = 446, *p* = 0.010) and (Mann-Whitney U = 2965, *p* = 0.004).

**Table 3:** Combined competencies score statistics by sector and assessment type

Assessment type & sector	Mean	Minimum	25th Percentile	Median	75th Percentile	Maximum
SA						
Hospital	5.35*	4.22	4.85	5.41	5.89	6.46
CHS	*5.39†*	3.81	4.96	5.53	5.83	6.63
CCA						
Hospital	5.81*	4.44	5.47	5.99	6.17	6.73
CHS	*5.69*†	4.00	5.36	5.75	5.99	6.24

*SA* Self-assessment; *CCA* Combined colleagues’ assessment; *CHS* Community Health Services; * & † significant differences between means as assessed by t-tests (*p* < 0.05)

Table 4 summarises the mean scores for each of the six core competencies from the SA and CCA by sector. Mean scores received for each core competency from both SA and CCA were all greater than five. However, the scores from SA and CCA and between competencies varied. Scores received from the CCA for each of the competencies were consistently higher than those from the SA. For hospital PPMs, the differences were all statistically significantly (p ranging from < 0.0005 to 0.045). For the CHS PPMs, the differences were only statistically significant for C2 (Resources) (*p* = 0.011) and C3 Knowledge (*p* < 0.0005). There were no significant variations in the SA mean competency scores for each core competency, each behavioural item and the combined competencies across sectors and organisations.

**Table 4:** Mean scores for six competencies by sector and assessment type (self and combined colleagues)

	Middle-Managers SA^a^	Hospital		CHS^c^	
		SA^a^	CCA^b^	SA^a^	CCA^b^
C1 (Evidence)	5.27	5.13	5.71^d^	5.20	5.57
C2 (Resources)	5.28	5.30	5.78^d^	5.26	5.58^d^
C3 (Knowledge)	5.16	5.20	5.98^d^	5.15	5.67^d^
C4 (Communications)	5.64	5.58	5.93^d^	5.66	5.77
C5 (Leadership)	5.56	5.46	5.81^d^	5.59	5.74
C6 (Change)	5.39	5.28	5.67^d^	5.43	5.60

^a^*SA* Self-assessment; ^b^*CCA* Combined colleagues’ assessment, ^c^*CHS* Community Health Services; ^d^Significant difference between SA and CCA scores by Mann-Whitney U tests; *p* < 0.05

Table 5 provides comparisons between the mean scores of the self-assessments and the mean scores of individual colleague assessments (supervisor, peer and report). Consistently, PPMs, supervisors and peers gave C4 (Communications) and C5 (Leadership) the highest scores amongst the six competencies. PPMs gave the lowest scores to C2 (Resources) and C3 (Knowledge) while supervisors and peers consistently gave the lowest scores to C1 (Evidence) and C6 (Change).

**Table 5:** Mean scores for six competencies by assessment type (all)

	Self-Assessment	Supervisor Assessment	Peer Assessment	Staff Assessment
C1 (Evidence)	5.27	5.44	5.73	5.74
C2 (Resources)	5.27	5.47	5.75	5.83
C3 (Knowledge)	5.16	5.52	5.79	5.93
C4 (Communications)	5.64	5.72	5.86	5.93
C5 (Leadership)	5.56	5.61	5.80	5.92
C6 (Change)	5.39	5.46	5.71	5.74
Combined competencies	5.38	5.55	5.75	5.86

In general, the mean competency scores received from supervisors, peer and reports were higher than the mean competency scores as self-assessed by the PPMs. The analytical tests for statistical significance of the differences between the mean self-assessment scores and the mean individual colleague assessment scores were as follows (details available in Table 6):

- Self-assessment versus supervisor scores: only the mean score for C3 (Knowledge) assessed by supervisors were statistically significantly higher than the mean self-assessment score;
- Self-assessment versus peer scores: means for all the six competencies assessed by peers were statistically significantly higher than the mean self-assessment score;
- Self-assessment versus report scores: means for all the six competencies assessed by reports were statistically significantly higher than the mean self-assessment score.

**Table 6:** Differences between mean self-assessment scores and mean colleague scores (supervisor (a), peer (b) and report (c)): t-tests for equality of means

	t	df	Sig. (2-tailed)	Mean Difference	95% CI of Difference	
					Lower	Upper
(a) Self- versus supervisor assessment						
Competency 1	−1.410	181	0.307	−0.176	−0.423	0.071
Competency 2	−1.682	181	0.094	−0.193	− 0.419	0.033
Competency 3	−3.166	181	0.002	− 0.357	− 0.579	− 0.134
Competency 4	− 0.888	181	0.375	− 0.087	− 0.280	0.106
Competency 5	−0.546	181	0.586	−0.057	−0.261	0.148
Competency 6	−0.526	181	0.600	−0.064	−0.302	0.175
Competencies combined	−1.705	181	0.090	−0.172	−0.371	0.027
(b) Self- versus peer assessment						
Competency 1	−4.453	178	< 0.0005	−0.461	−0.665	− 0.257
Competency 2	−4.673	178	< 0.0005	−0.471	−0.670	− 0.272
Competency 3	−6.177	178	< 0 < 0.0 < 0.0005	−0.621	−0.821	− 0.423
Competency 4	−2.578	178	0.011	−0.221	−0.389	− 0.052
Competency 5	−2.624	178	0.009	−0.242	−0.424	− 0.060
Competency 6	−3.169	178	0.002	−0.318	−0.516	− 0.120
Competencies combined	−4.260	178	< 0.0005	−0.375	−0.549	− 0.201
(c) Self- versus report assessment						
Competency 1	−4.067	175	< 0.0005	−0.476	−0.707	− 0.245
Competency 2	−4.909	175	< 0.000 < 0.0005	−0.552	−0.774	− 0.330
Competency 3	−6.940	175	< 0.000 < 0.0005	−0.761	−0.977	− 0.545
Competency 4	−2.752	175	0.007	−0.295	−0.507	− 0.084
Competency 5	−3.288	175	0.001	−0.360	−0.576	− 0.144
Competency 6	−2.960	175	0.004	−0.346	−0.576	− 0.115
Competencies combined	−4.577	175	< 0.0005	−0.476	−0.681	− 0.271

**Fig. 1:**
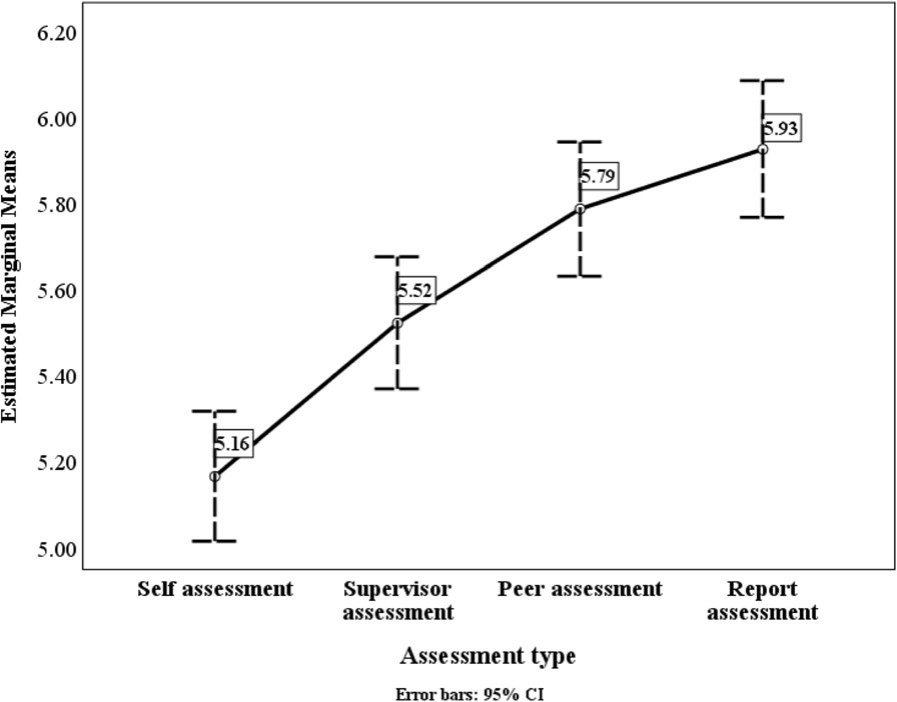
Mean scores for competency 3 (Knowledge) by assessment type (sectors combined)

Figure 1 shows the means scores for competency 3 (Knowledge) by assessment type. It is typical of all the analyses for the mean scores for competencies 1–6 and combined competencies.

The percentage of PPMs assessing themselves in low, medium and high score categories are provided in Table 7. Between 18 and 24% of managers assessed themselves as less than fully competent (< 5.0) for competencies **C1 (Evidence)**, **C2 (Resources**, **C3 (Knowledge)** and **C6 (Change).** Conversely, more PPMs assessed themselves fully competent or higher (scores 5.0–7.0) for competencies **C4 (Communications)** (91%) and **C5 (Leadership)** (89%). However, there were no significant differences in the distribution of competency groups between the management levels for all 79 behavioural items, the six competencies or the combined competencies scores.

**Table 7:** Percentage of managers at different competency levels (grouped)

Competency score group	Not yet fully competent		Competent (requiring vs not requiring guidance)		Proficient or higher
	< 3.0	3.0 - < 4.0	4.0 - < 5.0	5.0 - < 6.0	6.0–7.0
C1 (Evidence)	1%	2%	17%	34%	46%
C2 (Resources)	2%	3%	18%	29%	48%
C3 (Knowledge)	1%	3%	20%	35%	41%
C4 (Communications)	1%	1%	7%	30%	61%
C5 (Leadership)	1%	1%	9%	31%	58%
C6 (Change)	1%	2%	15%	31%	51%

Between three and 4 % of participants assessed themselves more than two standard deviations higher (between 1.8 and 2.3 points on the behavioural scale) than their supervisors, suggesting overconfidence by the participant (Table 7). Seventy-five to 80 % of these were CHS level III managers. Between 12 and 18% of participants assessed themselves more than one standard deviations higher (0.7 to 1.1 points) than their supervisors (Table 8). In addition, between 14 and 19% of supervisors assessed the participant more than one standard deviation higher (1.1 to 1.4 points) than the self-assessment.

**Table 8:** Number and percentage of self-assessment scores greater or less by more than two standard deviations or by more than one standard deviation compared to the supervisor scores

	C1 (%)	C2 (%)	C3 (%)	C4 (%)	C5 (%)	C6 (%)	Combined (%)
Greater than 2 SD	4 (4.4)	4 (4.4)	3 (3.3)	3 (3.3)	3 (3.3)	3 (3.3)	3 (3.3)
Less than 2 SD	1 (1.1)	1 (1.1)	1 (1.1)	1 (1.1)	0 (0)	0 (0)	0 (0)
Greater than 1 SD	16 (17.8)	14 (15.6)	16 (17.8)	13 (14.4)	14 (15.6)	11 (12.2)	14 (15.6)
Less than 1 SD	14 (15.6)	13 (14.4)	13 (14.4)	14 (15.6)	17 (18.9)	14 (15.6)	13 (14.4)

### Predictors of competency

Age was positively correlated with the means of the self-assessed six core competencies and the combined competency scores (correlation coefficients range: 0.25 to 0.35; *p* values ranging from 0.017 to 0.001). However, when adjusted for length of service as a middle level manager in a generalised linear model, age ceased to be significantly related (*p* values range: 0.56 to 0.186). Sex, length of service as a level 3/4 HSM, educational experience and membership of a professional institution were not statistically associated with the means of the self-assessed six competencies or the combined competencies both by the appropriate bivariate analyses, univariate analyses of variance and generalised linear modelling.

### Identification of potential behavioural items for improvement of individual primary participating managers (PPM)

There were great variations of scores between participants as self-assessed for the *individual behavioural items* in each of the competencies. Table 9 provides the mean scores from the self-assessments and the combined colleagues’ assessments for each of the competencies for three PPMs selected to represent low, average and high scorers. The table also presents the number of behavioural items that received a mean score less than five (less than fully competent) for each of the competencies. Behavioural items receiving a score less than five suggest that the PPM would benefit from further training. Where there are more items for the SA that are low scoring, this suggests a participant has underestimated their competency compared to their supervisor’s assessment. Where there were more low scoring items presented by the supervisor, the ‘high’ scoring participants had overestimated their competency compared to their supervisors’ assessments. This provides insight to their perception of competency, and can be used to explore where PPMs could improve their overall managerial competence, targeting professional development opportunities to the identified low scoring behavioural items. This demonstrates the applicability of the tool to individual managers to demonstrate competency gaps for targeted professional development.

**Table 9:** Results from three participating managers: competency means and number of items with scores less than five; self- and combined colleagues’ assessments

Competency	SA^a^	CCA^b^	SA^a^	CCA^b^	SA^a^	CCA^b^
	Low scorer (SA)		Average scorer (SA)		High scorer (SA)	
C1 Evidence						
*Mean score*	3.5	5.5	5.3	5.0	5.7	4.8
*# items < 5*	12	5	3	4	1	3
C2 Resources						
*Mean score*	4.2	5.6	5.2	5.5	6.2	4.8
*# items < 5*	13	5	2	3	0	4
C3 Knowledge						
*Mean score*	4.5	5.8	5.5	5.4	5.6	5.0
*# items < 5*	6	4	1	1	0	1
C4 Communications						
*Mean score*	4.7	6.2	5.5	4.9	6.5	5.6
*# items < 5*	7	4	1	3	0	2
C5 Leadership						
*Mean score*	4.5	6.1	5.4	5.0	6.3	5.1
*# items < 5*	6	3	1	2	0	3
C6 Change						
*Mean score*	4.0	6.0	5.3	4.9	6.1	4.9
*# items < 5*	8	5	3	4	1	5
Combined competencies						
*Mean score*	4.3	5.9	5.4	5.1	6.1	5.1
*# items < 5*	52	26	11	17	2	18

^a^*SA* Self-assessment, ^b^Combined colleagues’ assessment

## Discussion

This study has found the majority of middle-level HSMs in Victorian public hospital and community health centres demonstrate the core managerial competencies adequately and have some level of experience in managing health services. Both the SA and CCA mean scores from the MCAP 360° assessments were greater than five for all competencies measured. Furthermore, the proportion of HSMs that self-assessed scores above 5.0 ranged from 76 to 91%. According to the MCAP competency scale, a mean competency score 5.0 or higher indicates that the assessed participant is perceived as competent in applying the core behaviours in their managerial roles independently [27]. These results suggest that the competency level of the HSM workforce in Victoria is mostly appropriate for their roles and responsibilities.

Inevitably, there was some variation in competency assessment scores between managers employed across Victoria, with some scoring highly and some scoring low. The highest and the lowest mean combined competency scores from the self-assessment for hospital L4 managers were 6.46 and 4.22 respectively, while for CHS L3 managers they were 6.63 and 3.81 respectively. Discrimination between those excelling at core managerial responsibilities and those requiring support could be a powerful tool for healthcare organisations. Identifying top performing managers for “recruitment, competency management, learning and development, performance management, compensation and succession planning” is an important strategy in managing talent at the organisation level ([28], p41). An ability to identify the current workforce with an aptitude for managerial competency may lead to opportunities to recruit such people to positions of management strategically, and future research to explore the role of the MCAP in succession planning is warranted.

Two competencies were identified as having the largest proportion of managers demonstrating proficiency or higher (scores greater than six); number 6 (enabling and managing change) and number 4 (interpersonal, communication qualities, relationship management). The latter finding is not surprising, as the competency of *‘interpersonal, communication qualities and relationship management’* has long been identified as important for managers [23, 28–30] forming an integral component of professional and management training. Further, these competencies are also evident in clinical practice, with the Australian Health Practitioner Regulation Authority National Boards of Medicine, Nursing and the allied health disciplines all stipulating such behaviours in practice thresholds and standards (https://www.ahpra.gov.au/) The majority of the participants involved in this study were clinicians prior to commencing their HSM role, and 40% of participants still maintained membership of their clinical professional organisation. Staff transitioning from a clinically focussed role to a managerial role will have developed their competencies during their career as a clinician. Understanding how to support individuals transitioning from clinician to HSM role to transform their work competencies from the context of clinical practice to the context of HSM is research that should to be completed.

Apparent opportunities for improvement in competence of HSMs has been confirmed through this study, with 9–24% of participants scoring less than 5.0 in the self-assessment of a core competency. A mean score 4.0 or higher, but less than 5.0 indicates that the participant is competent but requires occasional guidance, suggesting a proportion of HSMs require support in their role. The competencies of *‘evidence-informed decision-making’* (20%)*, ‘resource management’* (23%) and *‘demonstrated knowledge of healthcare environment and the organisation’* (24%) were the three competencies that appear to require strengthening among participating HSMs, with the greatest proportion of participants scoring less than 5.0. Managers currently make limited use of the available evidence relevant to management and organisational practices [31, 32]. This finding may be related to the more recent addition of ‘*evidence-informed decision-making*’ being considered an essential competency for HSMs [22, 30]. A recent review of the formal Health Administration programs conducted within Australian universities, confirmed that *‘evidence-informed decision-making’* has not been widely accepted as a learning objective of the 13 Master of Health Administration degrees in Australia [10]. Evidence-based approaches to management can add to ‘substantive expertise’ ahead of ‘fads and popular tools’ of the time ([33], p84). Further emphasis on implementing an evidenced-based approach to management should be considered for HSM professional development curriculum and evaluation of the learning and impact on patient outcomes with development of competency in HSM should be completed.

Low performance may not necessarily be simply the capability or ‘fault’ of the individual receiving lower scores, as performance can be related to confidence or self-efficacy, insufficient opportunities to develop competency, low access to training and education opportunities, or unrealistic expectations of the team leader/manager or organisation [34]. The observed MCAP scores received from the CCA for each competency were consistently higher than those from the SA. Where an individual is lacking confidence, they may be more likely to rate themselves lower. The lower SA than CCA scores are consistent with previous literature that has demonstrated higher performance ratings are received from supervisors, peers and reports compared to self-ratings [34]. However, there was a subgroup of participants who assessed themselves higher than their supervisors (Table 8). This may be an example of the 'Dunnung-Kruger' effect which is a cognitive bias among people of low capability who asses their cometency as greater than it is [35, 36].This study did not measure potential moderating factors that may influence MCAP scores, and therefore future research is needed to explore the relationship between competency scores and confidence, experience and self-efficacy to understand reasons for low or high scores. From the organisation’s perspective, it is important to engage the low performing manager effectively to identify reasons for lower competency scores, such that skills and competencies requiring improvement can be targeted in professional development plans that are formulated to address the lower levels of observed competence.

### Variation of competency between sectors and organisations

In addition to variations between individuals within the same sector and managerial level, there are variations between mean scores received for six competencies between hospital and community health sector HSMs, with managerial strengths and weaknesses varying between sectors and organisations, in both the SA and CCA data. While competency requirements may be similar between sectors and across management levels, the actual demonstration of the competencies appears to vary, suggesting that the learning and professional develop needs differ. The design of ongoing management education and development should be context sensitive [37]. The constant reform of health systems across Australia and internationally has demonstrated the complexity of the HSM role [13, 14] and both practitioners and academics have called for a better understanding of the context of the HSM role in developing a capable, resilient and sustainable health management workforce [3]. The results from this study reaffirm this requirement and indicate that a ‘one size fits all’ approach to training is not likely to be effective. While system-wide strategies are important to set the training and development direction, separate consideration should be given to different management levels and groups from different sectors and from different organisations to contextualise the learning. Workforce development strategies need to be formulated in collaboration between policy-makers and managers at system, organisational and team levels, and future research evaluating the impact of such models of education, professional development, and training on health service provision is needed [38, 39].

### Limitations of the study

Although there is evidence linking management competency and individual performance [14], competency is context sensitive [37] and management competency may be influenced by various organisational factors. The participants were volunteers from organisations who agreed to participate and were not randomly selected for involvement. While this may not affect the internal validity of the study, it raises the question of the generalisability of the results to other populations of healthcare managers. Further evaluation beyond the Australian state of Victoria is needed to understand competency levels nationally.

Self-assessment is recognised as a potentially inaccurate method of assessing competence and may not reflect performance [40, 41, 42, 43], and this study measured competence by both self-assessment and colleague assessments subjective perspective only. Measurement of performance as a quantitative outcome is needed to confirm the relationship between self- or colleague assessed competency. While this study makes a strong addition to the literature and understanding competency levels of HSM in Australia, future studies should aim to examine manage competency levels and performance simultaneously.

### Practice implications for healthcare sectors and organisations

The exemplars provided in Table 6 illustrate the possible individual assessment data that can be provided through completion of the MCAP 360° assessment. The MCAP assessment not only provides the competence level for each of the core competencies, but also identifies behavioural items requiring improvements amongst each of the core competencies from self and colleagues’ perspectives. The five areas that the selected participating managers self-identified as requiring improvement were as follows:

- Use timely and appropriate questioning/investigation to identify the nature of a problem, issue or opportunity;
- Anticipate decision implementation problems/ impacts and develops and communicates appropriate contingency plans;
- Manage budgets in accordance with organisational objectives;
- Anticipate and plan for changes in policies affecting funding to the organisation/unit;
- Empower others to achieve goals.

This rich information about an individual manager supports the supervisor to design an individual professional development program, targeted at both specific behavioural items highlighted for development, but also specific behavioural items that are contextual to the organisation and specific to the role and tasks of the manager.

A focus on competency approaches such as the MCAP is not just about the performance of individual managers, but there is also the potential for such frameworks to be embedded within an organisation’s strategic direction. The ‘resource-based view of strategy emphasises the importance of an organisation’s core competencies in achieving sustainable advantage’ [38]. These core organisational competencies link to the internal capabilities of the organisation, which, in health care, substantially support the competence of the main resource, the workforce, and the human resource function that underpins it. Competency modelling, for which the MCAP provides the means, is an important innovation of process to assist organisations focus on job-related information and the skills required to manage others. Competency modelling may be useful to the agenda of secure job analysis, to distinguish performance of individuals, and to monitor and analyse how competencies might change at differing organisational levels or over time. Linking competencies of leaders, managers and employees to organisational objectives and strategies strengthens the potential use of the human resource capacity to respond to organisational sustainability [38, 44].

## Conclusion

The competency of the HSM workforce in Victoria, Australia, is appropriate for the roles and responsibilities the HSMs are expected to achieve. However, a relatively significant proportion of HSMs are demonstrating less than optimal competence that requires support or guidance suggesting that there is a learning professional development need. The most apparent area for development is the application of evidence-based decision making. Variations in competency are observed between hospital and community health sectors, indicating that professional development opportunities need to be contextualised for management level, sector and organisation. Further examination of HSM competency using the MCAP competency-based approach across other Australian States and in international contexts will provide a foundation to embed managerial competency framework into performance management and professional development of HSMs.

## Appendix

Six leadership and management competencies with some behaviour examples

### C1 Evidence-informed decision-making

C1.1 Use timely and appropriate questioning/investigation to identify the nature of a problem, issue or opportunity.

C1.6 Use evidence to question and improve existing practice and processes

C1.10 Set and use measures to evaluate decision outcomes

C1.11 Support and encourage colleagues and subordinates to use evidence to guide decision-making.

### C2 Operations, administration and resource management

C2.3 Interpret basic financial statements

C2.5 Develop budgets in accordance with organisational objectives

C2.8 Design and develop appropriate roles and reporting structure (across a range of areas) in accordance with organisational objectives

C2.13 Conduct regular two-way performance review & development discussions to support staff development

### C3 Demonstrated knowledge of healthcare environment and the organisation

C3.3 Demonstrate understanding of the roles of key stakeholders in health and how they interact

C3.4 Demonstrate understanding of the highly professionalised health workforce

C3.5 Apply relevant legislation and accountability frameworks specific to healthcare settings

C3.11 Effectively navigate organisational structures, roles and relationships in order to achieve work goals

### C4 Interpersonal, communication qualities and relationship management

C4.4 Engage confidently and constructively in verbal and non-verbal interactions with others

C4.6 Produce written reports/materials, which are appropriate for both audience and purpose

C4.7 Invest time and effort in working and engaging with stakeholders

C4.17 Show awareness of, and sensitivity to, the feelings of others

### C5 Leading people and organisation

C5.5 Balance the values and priorities of both organisation and profession(s)

C5.6 Lead, develop and evaluate performance to build an effective team

C5.8 Adapt leadership style to suit the situation

C5.9 Establish and maintain a personal and professional support network

### C6 Enabling and managing change

C6.1 Explain the need for change in an effective way

C6.2 Assess readiness for change and plans accordingly

C6.8 Evaluate the processes and outcomes of change

C6.9 Recognise and tolerate ambiguity

## Abbreviations

*ACHSM*: Australasian College of Health Service Managers
*CCA*: Combined colleague assessment
*CHC*: Community health centres
*HSM*: Health services managers
*MCAP LMCF*: Management Competency Assessment Partnership Leadership and Management Competency Framework
*MCAP*: Management Competency Assessment Partnership
*PPM*: Primary participating managers
*SA*: Self-assessment
